# The impact of hospital price and quality transparency tools on healthcare spending: a systematic review

**DOI:** 10.1186/s13561-022-00409-4

**Published:** 2022-12-14

**Authors:** Jinyang Chen, Marisa Miraldo

**Affiliations:** 1 School of Public Administration and Policy, Renmin University of China, No.59 Zhongguan Cun Avenue, Beijing, 100872 China; 2 Centre for Health Economics and Policy Innovation, Business School, Imperial College London, London, UK; 3 Department of Economics and Public Policy, Business School, Imperial College London, London, UK

**Keywords:** Price transparency, Quality transparency, Information disclosure, Healthcare spending, The reputation premium, Systematic review

## Abstract

**Background:**

Global spending on health was continuing to rise over the past 20 years. To reduce the growth rates, alleviate information asymmetry, and improve the efficiency of healthcare markets, global health systems have initiated price and quality transparency tools in the hospital industry in the last two decades.

**Objective:**

The objective of this review is to synthesize whether, to what extent, and how hospital price and quality transparency tools affected 1) the price of healthcare procedures and services, 2) the payments of consumers, and 3) the premium of health insurance plans bonding with hospital networks.

**Methods:**

A literature search of EMBASE, Web of Science, Econlit, Scopus, Pubmed, CINAHL, and PsychINFO was conducted, from inception to Oct 31, 2021. Reference lists and tracked citations of retrieved articles were hand-searched. Study characteristics were extracted, and included studies were scored through a risk of bias assessment framework. This systematic review was reported according to the PRISMA guidelines and registered in PROSPERO with registration No. CRD42022319070.

**Results:**

Of 2157 records identified, 18 studies met the inclusion criteria. Near 40 percent of studies focused on hospital quality transparency tools, and more than 90 percent of studies were from the US. Hospital price transparency reduced the price of laboratory and imaging tests except for office-visit services. Hospital quality transparency declined the level or growth rates of healthcare spending, while it adversely and significantly raised the price of healthcare services and consumers’ payment in higher-ranked or rated facilities, which was referred to as the reputation premium in the healthcare industry. Hospital quality transparency not only leveraged private insurers bonding with a higher-rated hospital network to increase premiums, but also induced their anticipated pricing behaviors.

**Conclusion:**

Hospital price and quality transparency was not effective as expected. Future research should explore the understudied consequences of hospital quality transparency programs, such as the reputation/rating premium and its policy intervention.

**Supplementary Information:**

The online version contains supplementary material available at 10.1186/s13561-022-00409-4.

## Introduction

In the past 20 years, healthcare spending was continuing to rise [1]. In order to reduce the growth rates of healthcare spending and strengthen consumer’s sovereignty, authorities and insurers have required hospitals to make price transparent [2]. At the same time, global health systems have also tracked, monitored, and publicly released hospital quality and performance metrics to alleviate information asymmetry on healthcare quality between providers and consumers [3, 4]. Overall, hospital price [5–8] and quality [9–14] transparency tools have been widely initiated around the world in the last two decades.

The policy effects of hospital price and quality transparency tools have not been thoroughly investigated. A stream of literature has estimated and synthesized [2, 3, 15–27] the impact of hospital price and quality transparency tools on provider’s provision and quality improvement behaviors [28–31] and consumer’s healthcare seeking behavior [32–38], while the relationship between hospital quality transparency and healthcare spending (i.e., the price of healthcare procedures and the payment of consumers) was overlooked to some extent. There were only 2 reviews synthesizing this relationship with a narrow scope (e.g., they omitted insurers’ contract provision and pricing behaviors), limited space, and dated evidence [22, 27]. To our knowledge, this is partly due to the fact that there was little evidence concerning this specific topic prior to 2015. With the growing number of related studies, it is possible to conduct this review to fill the knowledge gap and to inform health policy.

The objective of this review is to advance the understanding of the impact of hospital price and quality transparency tools on 1) the price of healthcare services and procedures, 2) the payment of consumers, and 3) the pricing behaviors of health insurance plans bonding with a hospital network. This review highlights the effects of hospital quality transparency tools that was overlooked by the existing reviews and proposes mechanisms.

## Methods

This review was conducted following the PRISMA guidelines [39, 40] and reported on studies categorized by types of interventions and outcomes, with summarizing the findings and the risk of bias for each included study. The reviewer anticipated that the interventions and outcomes would be too heterogeneous for meta-analysis and hence did not intend to meta-analyze the data. The study protocol was registered with PROSPERO with registration No. CRD42022319070.

### Search strategy and inclusion criteria

The reviewer searched SCOPUS, Econlit, Pubmed, Web of Science, EMBASE, PsycINFO, the Cumulative Index to Nursing and Allied Health Literature (CINAHL) for published literature starting from the database inception up to Oct 31, 2021. An example of a code used to search in SCOPUS is shown in Additional file 1. References from relevant articles were checked to see if any articles were omitted in the database searching process. Endnote X9 was used to import the search results, and duplicate articles were removed.

Articles were included if 1) the study design was observational or experimental; 2) the study examined the effects of hospital price and/or quality transparency tools; 3) the outcomes included any of the price of healthcare services, the payment of consumers, and the premium of health insurance plans bonding with a hospital network.

Articles were excluded if the studies 1) examined the effects of transparency tools not publicly available; 2) were non-quantitative; 3) were published in a language other than English; 4) solely evaluated the effects of financial incentive schemes such as reference pricing; 5) only described characteristics of hospital price and/or quality transparency tools.

### Data analysis and quality assessment

Following data was extracted from eligible papers: author and the year of publication; country; study design and the regression model; study sample; interventions; outcomes; overall conclusion.

All studies were evaluated according to the following quality assessment criteria: randomization, attrition rate, sample size, sample representativeness, the duration of transparency intervention, intervention and outcomes measurement (objective or subjective), confounders. The scoring framework consisted of 10 criteria, with scores 1 or 0. A final quality assessment score was generated by simply summing the points of each study. The total score was 10. Studies were considered low risk of bias if they had a score higher than 7, and high risk of bias if they had a score lower than 5. The risk of bias assessment result is provided in Additional file 2. Apart from this framework, the framework adapted from the Oxford Centre for Evidence-Based Medicine (OCEBM) [41, 42] by Carlo et al. (2020) [43] can also be employed to assess the quality of empirical studies in health economics and health policy. However, it cannot provide details on specific evaluating elements such as sample size and representativeness which is highly important for us to present a balanced review.

## Results

**Fig. 1:**
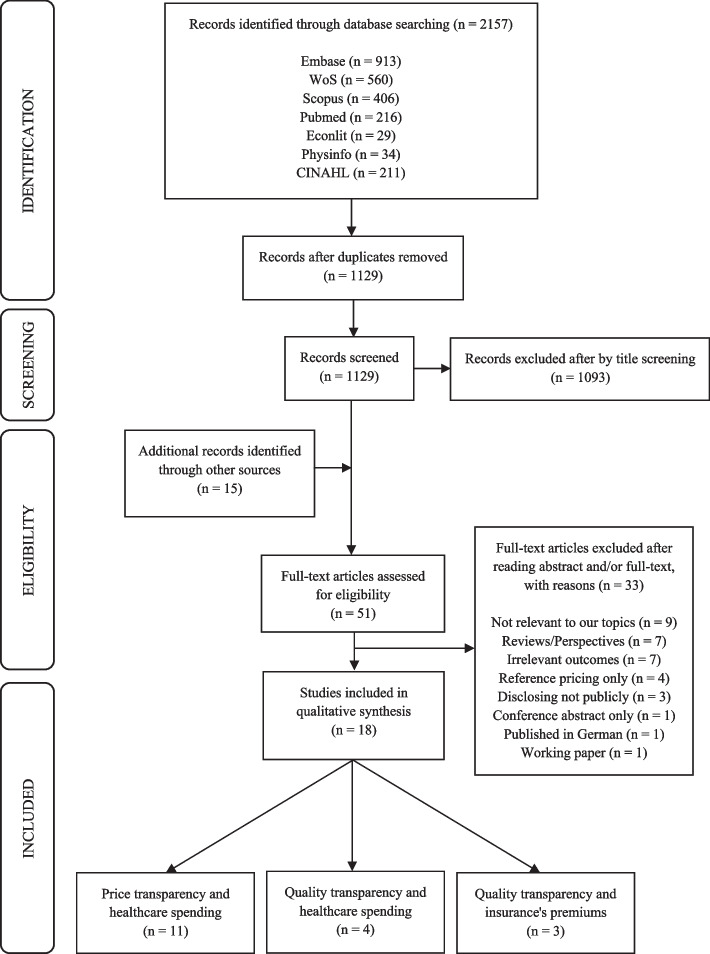
The PRISMA flow diagram

Eighteen studies of 2157 retrieved articles met the eligibility criteria (see Fig. 1 for the PRISMA flow diagram). As shown in Fig. 1, a total of 2157 articles were retrieved from seven databases. There were 1129 articles left after 1028 duplicates were removed. Following the title screening, 1093 articles were eliminated, leaving 36 articles. In addition, 15 articles were identified from reference lists. 33 of the 51 articles were eliminated after full-text screening because these studies were 1) not relevant to this review topic; 2) not quantitative analysis; 3) irrelevant to outcomes mentioned above; 4) not related to publicly available hospital transparency tools; 5) conference abstract only; 6) not been published in English; 7) working papers. 18 articles were included in our final synthesis. Articles were separated into three groups: 1) the impact of price transparency on healthcare spending; 2a) the impact of quality transparency on healthcare spending; and 2b) the impact of quality transparency on the premium of health insurance plans bonding with a hospital network.

A summary of 18 articles was provided in Table 1. There were 11 studies focusing on hospital price transparency tools, and 7 studies focused on hospital quality transparency tools of which 3 studies examined the impact of the hospital-network’s quality transparency on health insurance premiums. With regard to the source of evidence, 16 studies were conducted in the US and 2 studies were from Japan (*n* = 1) and China (*n* = 1), respectively. Study designs included randomized controlled trial^1^ (*n* = 2), quasi-experimental design (*n* = 13), and association analysis (*n* = 3). Quasi-experimental studies employed difference-in-differences (DID) and/or difference-in-differences-in-differences (DDD) (*n* = 11), regression discontinuity design (RDD) (*n* = 1) and other method (*n* = 1). Hospital transparency tools being examined consisted of government-initiated transparency tools (*n* = 8), private-initiated transparency tools by employers or private insurers (*n* = 7), and self-designed transparency tools by researchers (*n* = 3). Outcomes included the price of healthcare services and procedures (*n* = 10), the payment of consumers (*n* = 5), and the premium of health insurance plans bonding with a hospital network (*n* = 3).

**Table 1:** Study summary

Characteristics	Total number of studies (*n* = 18)	% of total studies
**Study theme**		
Hospital price transparency	11	61.1
Hospital quality transparency	7	38.9
**Study designs**		
RCT	2	11.1
Quasi-experimental	13	72.2
Association analysis	3	16.7
**Transparency programs**		
Government-initiated programs	8	44.4
Private insurers-initiated programs	7	38.9
Programs designed and intervened by researchers	3	16.7
**Outcomes**		
The price of healthcare services and procedures	10	55.6
The payment of patients	5	27.8
The premium of health insurance plan	3	16.7
**Study country**		
US	16	88.9
Japan	1	5.56
China	1	5.56
**Publication periods**		
prior to 2015	3	16.7
2016 to 2020	14	77.8
2021 to present	1	5.56

Study characteristics were displayed in Table 2. Following Table 2, a more detailed description and review were provided in the next two sections.

**Table 2:** Study characteristics

A**uthor/Pub Year/Citation**	**Study Country**	**Study Setting and Model**	**Study Sample**	**Interventions**	**Outcomes**	**Overall Conclusion**	**Risk of Bias Score**
**Panel A The effects of hospital price transparency**							
Wu et al. (2014) [44]	US	Quasi-experimental (pooled cross-sectional data), DID	105,637 patients	The implementation of a private insurer-initiated price transparency program	The change in average cost per imaging test	Negative	5
C. Whaley et al. (2014) [45]	US	Association analysis (pooled cross-sectional data), GLM	502,949 patients	Usage of the price transparency platform	Total payment amount (i.e., the sum of the patient and employer payments) at the procedure level (lab tests, imaging services, and clinician office visits)	Negative	7
Desai et al. (2016) [46]	US	Quasi-experimental (pooled cross-sectional data), Matching and DID	354,187 outpatients	Availability of the price transparency tool.	Annual outpatient spending, outpatient out-of-pocket spending, use rates of the tool.	Positive	7
Desai et al. (2017) [47]	US (California)	Quasi-experimental (pooled cross-sectional data), Matching and DID	843,533 beneficiaries	The implementation and usage of the price transparency tool	1) individual-level spending 2) average service-level price for lab tests, office visits, and imaging services.	No effect	6
Lieber (2017) [48]	US	Quasi-experimental (pooled cross-sectional data), DID	6208 employees (the unit of analysis rests on 387,774 procedures)	The sesearch behavior for price information through a given price transparency tool	The transacted price for procedures	Negative	5
C. Whaley et al. (2019) [49]	US	Quasi-experimental (pooled cross-sectional data), DID	1) 214,746 patients for laboratory tests (the unit of analysis rests on 2,443,211 claims records) 2) 32,363 patients for imaging tests (the unit of analysis rests on 37,750 claims records)	The implementation of an online price transparency (PT) tool in 2010, and a reference pricing program (RP) in 2011	The price of laboratory and imaging test	1) No effect, for PT only. 2) Negative, for PT and RP.	6
Brown (2019) [50]	US (New Hampshire)	Quasi-experimental (pooled cross-sectional data), DID	811,553 enrollees in New Hampshire	The implementation of an out-of-pocket price transparency website	Total visit price, patients’ out-of-pocket price, and insurers’ reimbursement price	Negative	6
Kobayashi et al. (2019) [51]	Japan (Tokyo)	Randomised controlled trial (pooled cross-sectional data), GLM	1053 outpatients	A randomly presented price list about outpatient healthcare services	Total payment amount	Positive	5
C. M. Whaley (2019) [52]	US	Quasi-experimental (longitudinal data), DID	93,974 office visit providers and 16,502 lab test providers	The staggered and nationwide diffusion of an online price transparency platform	The price for laboratory tests and office visit services	1) Negative for laboratory tests. 2) No effects for office visit services.	8
Carey & Dor (2020) [53]	US (New York and Florida)	Association analysis (longitudinal data), DID	8,616,184 inpatients in NY, and 9,802,568 inpatients in FL	The release of the CMS hospital charge report	The charges of hospital for inpatient services	Negative	4
Christensen et al. (2020) [54]	US	Quasi-experimental (pooled cross-sectional data), DID and DDD	1) 244,962 inpatients, and the unit of analysis rests on the charges and payments 2) 244,962 total payment records 3) 2,145,926 charge records	The disclosure date of price transparency website in each state	Charges and payments for 5 procedures	1) Negative for charge 2) No effects for payment	8
**Panel B The effects of hospital quality transparency**							
**Outcome 1 The price of healthcare services and the payment of consumers**							
Dor et al. (2015) [55]	US	Quasi-experimental (pooled cross-sectional data), DID	18,532 CABG inpatients and 54,301 PCI inpatients	The implementation of Hospital Compare mortality rankings	The transaction prices for CABG and PCI	1) Negative in the growth rates 2) BUT Positive in the price level	8
Huang & Hirth (2016) [56]	US (California, Florida, New York, Ohio, Texas)	Quasi-experimental (longitudinal data), DID	Around 7000 nursing facility	The differential ratings of nursing homes	The private-prices in nursing homes	1) Positive in the price level 2) Positive in the price and revenue differentials among higher- and lower-rated nursing homes	6
Liu et al. (2016) [57]	China (Qian Jiang City)	Randomised controlled trial (longitudinal data), DID	748,632 outpatient prescriptions	The public reporting (PR) about physicians’ prescribing information	Outpatients’ average expenditure	Negative	5
Dor et al. (2020) [58]	US	Quasi-experimental (pooled cross-sectional data), DID and DDD	20,773 CABG inpatients and 39,002 PCI inpatients	The implement of Hospital Compare, and hospitals’ differential rankings	The transaction prices for CABG and PCI	1) Negative in the price level 2) BUT Positive for higher-rated hospitals	8
**Outcome 2 The premium of health insurance plans bonding with hospital networks**							
McCarthy & Darden (2017) [59]	US	Quasi-experimental (pooled cross-sectional data), RDD	247,978 health plans	The introduction of the CMS quality star rating system for Medicare Advantage (MA) contracts	The premium of contracts	Positive for higher-rating contracts	9
McCarthy (2018) [60]	US	Quasi-experimental (pooled cross-sectional data), DID and FE model	311,571 health plans	The disclosure of CMS Medicare Advantage (MA) star rating program in period t + 1 or t + 2	The anticipated bids and premiums of health plans	1) Positive for lower-quality plans 2) Negative for higher-quality plans	9
Polsky & Wu (2021) [61]	US	Association analysis (cross-sectional data), LM	7706 health plans	A self-constructed hospital network quality factor	The premium of insurance plans	No effects	3

For Kobayashi et al. (2019) [51] and Liu et al. (2016) [57], these two studies did not meet the randomization requirement for RCT actually although they declared in the article that they are trial studies. Kobayashi et al. (2019) [51] didn't randomly assigned the participants to the hospital price transparency tool, and Liu et al. (2016) [57] didn't randomly assigned the quality transparency programs to primary care institutes

*DD* difference-in-differences, *DDD* difference-in-difference-in-differences, *GLM* generalized linear model, *FE* fixed-effects, *RDD* regression discontinuity design, *LM* linear model, *CABG* coronary artery bypass graft, *PCI* percutaneous coronary intervention

### The impact of price transparency on healthcare spending

In Panel A of Table 2 and 10 studies examined the impact of hospital price transparency on healthcare spending. These studies can be further divided into two categories: 1) 5 studies concerning the price of shoppable services (i.e., laboratory tests, image tests, and office visit services), and 2) 5 studies focusing on consumer’s healthcare payment and the costs of diagnosis groups.

### Outcome 1: the price of shoppable services

Overall, the price of shoppable services was significantly reduced by hospital price transparency tools. In terms of laboratory tests, the implementation of hospital price transparency tools caused 1–4% price reduction [52], while this effect was more (or only) significant in sub-samples who searched for price information in reality (i.e., the compliers in econometrics), with a reduction raised to 13.93% [45]. When price transparency tools such as an online price searching website were combined with a financial incentive tool such as reference pricing programs, there was a more sizeable price reduction, approximately 27% [49]. Apart from the above negatively significant findings, the policy effects of hospital price transparency were found to be insignificant [47] or positively significant in Japan [51]. With regard to imaging tests, the findings were the same as above [44, 45, 47] regarding whether hospital price transparency tools were implemented alone or not [49]. A positive relationship was reported in Japan [51]. As for visit services, the existing evidence did not provide any significant results [45, 47, 52].

Although the evidence seems to suggest mixed findings the study that reports positive effects in Japan had a high risk of bias [51]. In particular, the study sample was not representative. The outpatients in this study might have a relative high-income, and their price elasticity of demand might be smaller than the population. Therefore, the possibility of being driven to shop lower-priced healthcare services for those high-income outpatients would be low and a downward effect cannot be found. Although it was a field experimental study, there was no random assignment to treatment and therefore one can not rule out sample selection biases concerns. Moreover, the duration of the intervention only lasted two weeks and thus it is plausible that the length of duration did not allow enough time to inform outpatients (see Additional file 2).

### Outcome 2: the payment of patients and the cost of diagnosis groups

The payment of consumers decreased significantly in general, after the implementation of hospital price transparency tools. For those who had access to and actually used price transparency tools, the average total payments decreased by 1.6% [48] and it was referred as a selection effect from the demand side [50]. If the demand-side effect is weak or does not exist, the healthcare spending could also be reduced. For example, when hospital price transparency such as the HealthCost website in New Hampshire was available to the entire market, the out-of-pocket spending among all inpatients was declined by 5%, which was driven by the price competition on the supply side rather than the selection effect on the demand side as many inpatients did not use this price transparency website [50]. This downward effect appears not only at the patient level but also at the disease level. For example, the costs of publicly disclosed diagnosis-related groups (DRGs) grew lower than undisclosed DRGs by 4–9% in New York hospitals, and by 2–8% in Florida hospitals [53].

The reduction on the price of healthcare procedures and the cost of diagnosis groups above did not imply patients would pay less, because hospitals in the US decreased discounts simultaneously thus disconnecting decreases in the price of healthcare procedures and/or services from any changes in consumer’s payment [54].

### The impact of quality transparency on healthcare spending

#### Outcome 1: the price of healthcare services and the payment of consumers

All studies reviewed found a negative effect on average. As shown in Panel B of Table 2, hospital quality transparency did decrease the overall level and/or the growth rates of healthcare prices on average. Dor et al. (2015) [55] estimated the impact of Hospital Compare (HC) quality reporting program initiated by the U.S. Centers for Medicare & Medicaid Services (CMS) on transaction prices for coronary artery bypass graft (CABG) and percutaneous coronary intervention (PCI). Although the price continued to rise after introducing HC quality scores, the growth rates were significantly lower in the treated states than in the control states. Similarly, Liu et al. (2016) [57] conducted a matched-pair cluster-randomized trial in Qian Jiang city of China, and the results suggested that primary healthcare facilities’ performance transparency decreased patients’ healthcare spending by 5.1% [57]. While the former study had a low risk of bias score, the second study was conducted in a small area (i.e., Qian Jiang city in China) [57]. Although the macro-society and economy indicators in this assessed small area were generally in line with the average level across all counties in China, the sample was not representative of Chinese population. Moreover, there was no random assignment of hospital quality transparency to the primary healthcare facilities in this trial study (see Additional file 2).

Findings were opposite if the studies focused on higher-ranked/rated facilities and explored the price differentiation across higher- and lower-ranked/rated facilities. Specifically, although HC exerted downward pressure on price, hospitals with “above-average” ratings still captured higher prices, thereby partially or fully offsetting the policy effect [58]. Similarly, in the nursing home industry, Huang and Hirth (2016) [56] found an upward price differential between top- and bottom-ranked facilities, and top-ranked facilities had a 4.8-6.0% average increase over the bottom-ranked facilities. Given the evidence listed above, the existing study argued that ceteris paribus higher-ranked or rated facilities may capture the reputation premium via hospital quality and performance transparency programs [58], which did not receive close attention until now.

If the reputation premium was captured by higher-ranked or rated facilities indeed [56, 58, 62], the welfare analysis could be more complex. Welfare improvement due to a price reduction in lower-ranked/rated facilities might be somewhat offset by increased healthcare price among higher-ranked/rated facilities [60], and hospital quality transparency tools may not have increased consumers’ welfare. In this topic, the evidence is limited to the US, and it is vital to complement more evidence from other public-featured healthcare systems [19, 62] and developing countries [62].

### Outcome 2: the premium of health insurance plans bonding with a hospital network

Hospital quality transparency tools significantly increased the premium of health insurance plans bonding with a high-quality hospital network and induced their forward-looking pricing behaviors. McCarthy and Darden (2017) [59] measured insurers’ pricing behavior in responding to the CMS five-star rating scheme, and they found that contracts with higher star ratings in 2009 significantly raised their average monthly premiums considerably in 2010, compared to insurance plans just below their respective threshold values, with hikes of more than $26 per month for 3.5 and 4-star contracts. Besides, McCarthy (2018) [60] further examined the presence of forward-looking behavior under the same research context and found that, prior to quality disclosure, the price was decreased in high-quality insurers but increased in low-quality insurers adversely. Both studies have low risk of biases scores (see Additional file 2). Although Polsky and Wu (2021) [61] did not find a significant association between the premium of contracts and the quality of hospital networks, their study design has a high risk of bias (see Additional file 2).

## Discussion

### Explanations for the heterogeneity of effects

The findings varied notably across countries and periods, especially for the effects of hospital price transparency tools. Some explanatory factors have been listed in the previous systematic review, including 1) low usage of price transparency tools resulting in limited population-wide effects, 2) diverse methods of initiating the price transparency tool, and 3) disparities in healthcare services assessed [2]. However, some critical aspects were omitted, and this review might partially bridge this gap.

On the one hand, the heterogeneity naturally comes from the differences on price regulation policies across study settings. After introducing the hospital price transparency program, the payment of outpatients were increased in Japan [51]. Although this study had a high risk of bias and therefore one cannot rule out biased estimates, results could also be attributed to the fact that the Japanese government initiated a “national fee schedule” to establish the unit price of healthcare services and patients therefore had no incentive to shop around since prices were the same among different providers [51].

On the other hand, the effectiveness of the hospital price transparency may largely depend on its design features. Applying price transparency tools, such as price searching website, jointly with reference pricing programs could yield better results than employing the former alone. Without the reference pricing program, patients still need to navigate the distribution of provider prices even if they have been informed by price transparency tools about the price of specific providers. In such a case, if the searching cost is high, the financial incentives from a high-deductible insurance plan may not be strong enough to drive patients’ searching and selection behavior [49]. On the contrary, when the transparency mechanism is designed ingeniously, the financial and informational obstacles to price shopping are both tackled [46, 49] and patients are not only able to find many low-cost providers but also are directed towards a few low-priced providers by reference pricing programs [49]. That is why the effects of applying hospital price transparency tools alone are generally smaller than that of combining price transparency tools jointly with reference pricing programs.

### How transparency affects healthcare spending

Clarifying potential mechanisms is vital for an in-depth understanding of policy effects. In previous systematic reviews, it had been well-documented that consumers did respond to hospital price and quality transparency tools through having access to or searching for disclosed information [45–49], choosing providers [44, 46, 47, 51], and switching health insurance plans [59, 60]. Although the demand-side mechanisms listed here are critical, supply-side mechanisms are also important but overlooked.

First, hospital competition is undoubtedly the most important and most frequently mentioned mechanism, while its welfare consequences could be less straightforward than expectations. As informed by Huang and Hirth (2016), in less concentrated markets, hospital price and quality transparency tools may lead prices to reflect the marginal cost better and sort consumers based on their willingness-to-pay simultaneously [56]. On the contrary, in highly concentrated markets, hospital price and quality transparency induced an increased price differentiation across higher- and lower-performance hospitals, and this price differentiation may predominantly reflect the willingness-to-pay rather than the marginal cost of healthcare production. In such a case, the surplus was transferred from consumers to providers [56].

Second, in the healthcare industry, providers respond to price and quality transparency tools not only for competition, but also for reputation. For instance, patients’ healthcare payment in facilities not being affected by transparency programs declined the same as other treated facilities [44, 50]; following price transparency regulation, a larger price reduction was found in nonprofit/state-owned and church-affiliated hospitals serving a relatively high-proportion of low-income patients and hospitals facing higher intensity of public scrutiny of healthcare costs (e.g., the number of Google searches for the term “healthcare costs” and the reputation pressure from local newspapers) [54]. Since reputation (intrinsic incentive) is as crucial as competition (extrinsic incentive), it would be beneficial for health policy makers to clearly separate and compare the relative importance of competition and reputation in controlling the growth of healthcare spending, which can enrich the policy toolbox. However, the evidence is highly limited until now.

Third, on top of competition and reputation incentives, the bargaining process matters also. In healthcare systems predominantly financed by private health insurance, formal pay-for-performance for hospitals are less applicable, and higher-performance providers’ compensation is mainly left to market forces such as the bargaining process between providers and purchasers [58]. In this process, the implementation of hospital quality and performance transparency dynamically redistributes the bargaining power across purchasers and providers while the effect of this dynamic bargaining process within the context of hospital quality transparency is still unclear [55, 56, 58].

Fourth, hospitals did take advantage of the low usage of price transparency tools to strategically raise price. The low usage of price transparency tools should also be seen as a critical supply-side mechanism. If most patients do not care about disclosed price information, providers in less competitive markets with lower price would be inspired to match higher-priced peers, thereby reducing price variance but increasing overall price level [26, 53]. This hypothesis was examined in 2020 for the first time, and the evidence showed that, after the cost information of DRG was publicly disclosed, hospitals in the lowest quintile of cost distribution had the highest percent increase in the cost of DRGs [53]. It is an understudied area left for further study.

## Conclusion

Hospital price and quality transparency is not effective as expected. Based on limited evidence from the US, hospital price transparency reduced the price of laboratory and imaging tests while had an insignificant impact on consumers’ total payment due to the fact that the usage of transparency tools was low and hospital decreased discounts simultaneously. Hospital quality transparency generally declined the price of healthcare procedures. However, for those higher-ranked/rated hospitals and nursing homes the price was raised and consumer’s private payment was increased, which indicates that higher-ranked/rated facilities might be able to capture the reputation premium via quality and price transparency programs. Hospital price and quality transparency also increased the premium of health insurance plans bonding with a high-quality hospital network and induced their forward-looking pricing behaviors.

This review has three potential contributions. First, this review meets the need for clarity on the impact of hospital transparency tools on healthcare spending [63], highlighted as a key research gap in the literature [16, 22, 25, 26]. Second, this review identifies disparities across studies within the same research topic and proposes explanations overlooked by previous reviews. Third, this review further clarifies the omitted mechanism through which hospital price and quality transparency tools affect healthcare spending.

Certain limitations should be noted also. First, the majority of the studies included in this review (16 of 18) were conducted in the US, and the external validity of our conclusion might be restricted. Second, the number of studies being identified in this study is relatively small due to the tight searching queries and a relative short time window. Third, the overwhelming majority of studies revealed statistically significant effects, which might be attributed to the fact that authors and publishers were unwilling to publish research indicating an insignificant relationship or impact. In the future, more research should focus on the understudied impact of hospital quality transparency programs such as hospital rating or ranking scheme on healthcare spending.

Despite these caveats, this review promotes a comprehensive understanding of whether, the extent to which, and how hospital price and quality transparency tools shaped the economic behaviors of both providers, consumers, and purchasers (insurers), and that is instrumental for the design of policies and interventions in health systems to promote efficiency, population health and welfare.

## Supplementary Information

**Additional file 1. **Searching strategy for Scopus.

**Additional file 2.** Risk of bias assessment table.

## Data Availability

Data sharing is not applicable to this article as no datasets were generated during the current study.

## Abbreviations

CABG: Coronary artery bypass graft
CMS: The U.S. Centers for Medicare & Medicaid Services
DD: Difference-in-differences
DDD: Difference-in-difference-in-differences:DRG:Diagnosis related groups
FE: Fixed-effects
GLM: Generalized linear model
HC: Hospital compare
LM: Linear model
OCEBM: The Oxford Centre for Evidence-Based Medicine
PCI: Percutaneous coronary intervention
RCT: Randomized controlled trial
RDD: Regression discontinuity design
US: The united states of America

## Footnotes

1 These two studies did not meet the randomization requirement for RCT actually. The participants were not randomly assigned to the treatment and the quality transparency program was not randomly assigned to the studied primary care institutes.
