# Supplementary material for: The impact of hospital price and quality transparency tools on healthcare spending: a systematic review
Source: Health Econ Rev. 2022 Dec 14;12:62. doi: 10.1186/s13561-022-00409-4 (PMC9749158; doi:10.1186/s13561-022-00409-4)
Supplement: Supplementary file 1 — Additional file 1. Searching strategy for Scopus. [file 13561_2022_409_MOESM1_ESM.docx]

**Additional file 1** Searching strategy for Scopus

|  | Searches | Results |
| --- | --- | --- |
| 1 | TITLE (quality) AND TITLE (disclos* OR transparen* OR report* OR tool* OR information OR variation OR improv* OR ascend* OR descend* OR increas* OR decreas*) | 89086 |
| 2 | TITLE (pric*) AND TITLE (disclos* OR transparen* OR report* OR tool* OR information OR variation OR improv* OR ascend* OR descend* OR increas* OR decreas*) | 6767 |
| 3 | TITLE (hospital*) AND TITLE (rank* OR rat* OR compar* OR league OR report*) | 27806 |
| 4 | OR/1-3 | 123078 |
| 5 | 4 AND TITLE (impact OR effect* OR affect* OR associat* OR experiment* OR trial* OR evidence) | 15018 |
| 6 | 5 AND TITLE (pric* OR charg* OR cost* OR bill* OR fee* OR expense* OR expenditure* OR insurance OR plan* OR premium*) | 1898 |
| 7 | 6 AND TITLE-ABS-KEY (hospital*) | 406 |
