# Supplementary material for: The impact of hospital price and quality transparency tools on healthcare spending: a systematic review
Source: Health Econ Rev. 2022 Dec 14;12:62. doi: 10.1186/s13561-022-00409-4 (PMC9749158; doi:10.1186/s13561-022-00409-4)
Supplement: Supplementary file 2 — Additional file 2. Risk of bias assessment table. [file 13561_2022_409_MOESM2_ESM.docx]

**Additional file 2** Risk of bias assessment table

| Author/year/ref | Was the study randomized controlled trial (RCT) (yes =1, no = 0) | Did the study account for attrition rates? (for retrospective/quasi-experimental studies, accounting for the take up is 1, otherwise 0) | Does the study have an appropriate (large) sample size? (yes =1, no = 0) | Does the study have a representative sample? (yes =1, no = 0) | Was the intervention long enough?  (more than 6 months is 1; 0 otherwise) | Was the intervention objectively measured? (the searching behavior and the usage of information are 1, and the transparency policy implementation is 1 also; 0 otherwise) | Were the outcomes objectively measured? (the price and the payment as objective; all self-reported outcomes as not objective) | Were all important confounding factors considered?  (1 if accounts for characteristics of patients, facilities, and markets; 0 if only some of those) | Are the results of the treatment effect well reported and is the analysis coherent? (yes =1, no = 0) | Are the estimates of the treatment effect precise? (yes =1, no = 0) | **Score** |
| --- | --- | --- | --- | --- | --- | --- | --- | --- | --- | --- | --- |
| Wu et al. (2014) [44] | 0 | 0 | 1 | 0 | 1 | 1 | 1 | 0 | 1 | 0 | 5 |
| C. Whaley et al. (2014) [45] | 0 | 0 | 1 | 0 | 1 | 1 | 1 | 1 | 1 | 1 | 7 |
| Desai et al. (2016) [46] | 0 | 1 | 1 | 0 | 1 | 1 | 1 | 1 | 1 | 0 | 7 |
| Desai et al. (2017) [47] | 0 | 0 | 1 | 0 | 1 | 1 | 1 | 1 | 1 | 0 | 6 |
| Lieber (2017) [48] | 0 | 1 | 1 | 0 | 0 | 1 | 1 | 0 | 1 | 0 | 5 |
| C. Whaley et al. (2019) [49] | 0 | 0 | 1 | 0 | 1 | 1 | 1 | 0 | 1 | 1 | 6 |
| Brown (2019) [50] | 0 | 1 | 1 | 0 | 1 | 1 | 1 | 0 | 1 | 0 | 6 |
| Kobayashi et al. (2019) [51] | 0a | 0 | 1 | 0 | 0 | 1 | 1 | 1 | 1 | 0 | 5 |
| C. M. Whaley (2019) [52] | 0 | 1 | 1 | 1 | 1 | 1 | 1 | 1 | 1 | 0 | 8 |
| Carey & Dor (2020) [53] | 0 | 0 | 1 | 0 | 1 | 1 | 1 | 0 | 0 | 0 | 4 |
| Christensen et al. (2020) [54] | 0 | 0 | 1 | 1 | 1 | 1 | 1 | 1 | 1 | 1 | 8 |
| Dor et al. (2015) [55] | 0 | 1 | 1 | 1 | 1 | 1 | 1 | 1 | 1 | 0 | 8 |
| Huang & Hirth (2016) [56] | 0 | 0 | 1 | 0 | 1 | 1 | 1 | 1 | 1 | 0 | 6 |
| Liu et al. (2016) [57] | 0b | 0 | 1 | 0 | 1 | 1 | 1 | 0 | 0 | 1 | 5 |
| Dor et al. (2020) [58] | 0 | 1 | 1 | 1 | 1 | 1 | 1 | 1 | 1 | 0 | 8 |
| McCarthy & Darden (2017) [59] | 0 | 1 | 1 | 1 | 1 | 1 | 1 | 1 | 1 | 1 | 9 |
| McCarthy (2018) [60] | 0 | 1 | 1 | 1 | 1 | 1 | 1 | 1 | 1 | 1 | 9 |
| Polsky & Wu (2021) [61] | 0 | 0 | 1 | 1 | 0 | 0c | 1 | 0 | 0 | 0 | 3 |

a. The participants were not randomly exposed to the hospital price transparency program.

b. The quality transparency intervention was not randomly assigned to the primary care institutes.

c. The quality factors were self-constructed by researchers.
